# Prognostic value of the aggregate index of systemic inflammation and controlling nutritional status score for all-cause mortality in patients undergoing maintenance hemodialysis: a retrospective cohort study

**DOI:** 10.3389/fmed.2026.1815153

**Published:** 2026-06-18

**Authors:** Haitao Bai, Shuang Cheng, Dan Lv, Yilan Liang, Wenli Chen

**Affiliations:** 1 Department of Nephrology, The Central Hospital of Wuhan, Tongji Medical College, Huazhong University of Science and Technology, Wuhan, China; 2 Department of Clinical Nutrition, The Central Hospital of Wuhan, Tongji Medical College, Huazhong University of Science and Technology, Wuhan, China

**Keywords:** aggregate index of systemic inflammation, all-cause mortality, cohort study, controlling nutritional status score, maintenance hemodialysis, predictive factors

## Abstract

**Background:**

This study investigated the prognostic relevance of the Controlling Nutritional Status (CONUT) score and the Aggregate Index of Systemic Inflammation (AISI) for predicting all-cause mortality (ACM) in patients undergoing maintenance hemodialysis (MHD). Furthermore, the study aimed to identify independent prognostic indicators to aid clinical risk categorization and tailored therapeutic interventions.

**Methods:**

A retrospective cohort study was performed with a follow-up duration of 5 years. Extensive demographic data and clinical laboratory results were gathered, and patients were followed until December 2024, with ACM set as the principal endpoint. The predictive capabilities of the CONUT score and AISI for ACM among MHD patients were evaluated by receiver operating characteristic (ROC) curve analysis. Survival outcomes were depicted through Kaplan–Meier curves, and Cox proportional hazards regression was utilized to ascertain significant prognostic factors for five-year ACM.

**Results:**

In total, 614 patients on MHD were enrolled. ROC analysis yielded areas under the curve (AUC) of 0.704 for the CONUT score and 0.694 for AISI, with optimal threshold values identified at 3.5 and 306.100, respectively. Patients exhibiting AISI below 306.100 had markedly better cumulative survival rates (*χ*^2^ = 48.085, *p* < 0.001). Similarly, patients with CONUT scores equal to or greater than 3.5 displayed significantly reduced cumulative survival compared to those scoring below 3.5 (*χ*^2^ = 65.147, *p* < 0.001). Multivariate Cox regression analyses demonstrated that central venous catheter (CVC) utilization, CONUT score ≥ 3.5, AISI ≥ 306.100, and elevated aspartate aminotransferase (AST) levels were independently associated with increased five-year ACM (*p* < 0.05). Conversely, being female and exhibiting elevated concentrations of hemoglobin (HGB), urea, and creatinine were protective against mortality (*p* < 0.05).

**Conclusion:**

Both AISI and the CONUT score provide significant prognostic information for predicting ACM in patients undergoing MHD. Their combined use constitutes a robust, accessible tool for identifying high-risk individuals in this population.

## Introduction

Chronic kidney disease (CKD) has emerged as a critical global public health issue, imposing substantial healthcare, economic, and societal burdens worldwide. Current estimates indicate that approximately 788 million adults aged 20 years or older are affected by CKD globally, corresponding to an age-standardized prevalence rate of approximately 14.2%. Specifically, China alone contributes significantly to this global health challenge, with an estimated 152 million individuals diagnosed with CKD (1). As CKD progresses, patients experience gradual deterioration in renal function, and many ultimately advance to end-stage renal disease (ESRD), a condition that requires renal replacement therapy (RRT) to sustain life. The primary therapeutic approaches for managing ESRD include hemodialysis (HD), peritoneal dialysis (PD), and kidney transplantation, with HD remaining the most widely utilized modality due to its accessibility and relative ease of implementation. Data from the Chinese Society of Nephrology indicate that as of 2024, the population of patients undergoing HD in mainland China has reached 1,027,267 individuals, highlighting the immense clinical and public health challenge associated with managing this patient cohort.

Patients undergoing MHD frequently encounter clinical complications, notably malnutrition and chronic systemic inflammation. These conditions are recognized for significantly contributing to increased morbidity, mortality rates, and extended hospital stays (2). Consequently, the early identification and targeted management of inflammation represent pivotal strategies for enhancing clinical outcomes and overall patient survival. Nonetheless, routine clinical integration of conventional inflammatory biomarkers remains limited due to high testing costs, technical complexity, and the requirement for specialized laboratory infrastructure. In this context, the AISI, a composite inflammatory biomarker that is both cost-effective and easily obtainable from routine blood tests, has been proposed as a potentially superior clinical indicator. Unlike single inflammatory markers, the AISI offers a more comprehensive reflection of systemic inflammatory responses, capturing the intricate interplay among neutrophils, lymphocytes, monocytes, and platelets. Prior studies have validated the prognostic value of the AISI across diverse patient populations, demonstrating its utility in predicting clinical outcomes in diseases such as diabetes mellitus (3), various malignancies (4), psychiatric disorders including depression (5), and PD populations (6). Mechanistically, elevated AISI values denote a heightened inflammatory milieu characterized by increased neutrophil and monocyte activity, elevated platelet counts, and diminished lymphocyte levels. Collectively, these hematologic shifts indicate aberrant immune activation, intensified inflammatory cascades, and compromised immune defense mechanisms, ultimately predisposing patients to adverse clinical events and increased mortality risks.

Concurrently, nutritional status assessment remains critical in managing MHD patients, with nutritional deficiencies recognized as potent predictors of adverse clinical outcomes. The CONUT score has emerged as a validated, objective nutritional assessment tool, integrating three key serum-derived parameters: total cholesterol (TC), serum albumin (Alb), and total lymphocyte count (LYM). Each parameter within the CONUT scoring system is individually evaluated and assigned scores ranging from 0 to 2 points according to established clinical thresholds, with higher aggregate scores correlating with progressively poorer nutritional states. Previous clinical investigations have consistently confirmed the CONUT score’s predictive value in various medical contexts, including diabetes (7), heart failure (8), stroke (9), and cancer patient populations (10).

In routine clinical settings, both the AISI and the CONUT score represent accessible, cost-effective, and easily implementable tools, potentially facilitating comprehensive patient evaluation. Nevertheless, evidence regarding their combined predictive capacity in patients undergoing MHD remains notably sparse. To address this research gap, the current study was designed to investigate the relationship between the AISI and the CONUT score and their combined impact on the five-year ACM among individuals receiving MHD. We hypothesized that combining these two indices would yield superior prognostic accuracy for five-year ACM compared with using either index individually, given their complementary roles in assessing inflammatory and nutritional risks. Additionally, the study sought to elucidate independent mortality predictors within this patient population. By providing clarity on these associations, this research aims to establish robust clinical evidence to support improved patient risk stratification, facilitate targeted therapeutic interventions, and ultimately enhance survival outcomes for MHD patients.

## Methods

### Study design and population

This observational cohort investigation, conducted retrospectively at a single medical institution, enrolled participants who underwent MHD from three blood purification units within Wuhan Central Hospital beginning in January 2020. Inclusion criteria: (1) aged at least 18 years, (2) received consistent HD treatment for a minimum duration of 3 months accompanied by reliable follow-up records, and (3) had comprehensive clinical data available. Exclusion criteria encompassed: (1) initiation of dialysis due to acute renal failure, (2) use of peritoneal dialysis (PD) or transitioning from PD to HD, (3) irregular or intermittent dialysis schedules, and (4) presence of severe infection, liver failure, malignant tumors, or hematological disorders. Ethical approval was granted by the Ethics Committee at Wuhan Central Hospital (approval reference WHZXKYL2022-112-01).

### Data collection and follow-up

Clinical laboratory results, demographic details, and dialysis-related parameters were obtained from the hospital’s electronic records by trained personnel. Laboratory measurements were performed quarterly throughout the follow-up period, and the mean values of all available measurements prior to death or censoring were included in the analyses, following the methodology described by Kang et al. (11). This strategy minimized short-term fluctuations and more accurately reflected the long-term inflammatory and nutritional status of patients undergoing MHD. Laboratory values obtained during hospitalization or active infection episodes were excluded to avoid confounding from acute inflammatory responses. The AISI was calculated using peripheral blood cell counts according to the following formula: neutrophil count × monocyte count × platelet count / lymphocyte count. Blood Alb, TC, and LYM were used to determine the CONUT score. Total scores varied from 0 to 9, with parameters being graded from 0 to 3. A normal nutritional condition was indicated by a score of 0, mild to moderate malnutrition by a score of 1–2, and severe malnutrition by a score of ≥3.

### Follow-up and dialysis protocol

Follow-up concluded on December 31, 2024. Patients were censored upon conversion to PD, kidney transplantation, or transfer to another facility. All patients received HD treatment 2–3 times weekly, with each session lasting 4 h. Standard bicarbonate HD was performed using Fresenius 4008S or 5008S (Germany) or Braun dialysis machines, combined with high-flux disposable polysulfone dialyzers.

### Statistical analysis

Variables were presented as means ± standard deviations (SDs), and categorical variables as frequencies and percentages. Kaplan–Meier analysis was applied to estimate survival rates, and survival differences between groups were tested using log-rank statistics. Hazard ratios (HRs) along with corresponding 95% confidence intervals (CIs) were computed. Variables showing initial statistical significance underwent subsequent analyses to determine independent risk factors. Restricted cubic splines (RCS) with knots at the 25th, 50th, 75th, and 95th percentiles were used to evaluate potential nonlinear relationships between AISI and mortality. ROC curves were constructed to assess predictive accuracy, quantified using the area under the curve (AUC). Optimal thresholds for AISI and CONUT were identified by maximizing the Youden index, and the sensitivity and specificity of these indices for predicting ACM were subsequently evaluated. Internal validation of the predictive performance was performed using bootstrap resampling with 1,000 repetitions. Survival curves stratified by high or low values of AISI and CONUT were compared using log-rank tests. Cox proportional hazard models were constructed to identify independent factors associated with five-year ACM in MHD patients. To prevent multicollinearity, individual parameters included in the AISI and CONUT scores were excluded from the multivariable analysis. Multicollinearity diagnostics were performed using tolerance values and variance inflation factors (VIFs), with a VIF < 5 and tolerance > 0.2 indicating no substantial multicollinearity. To account for potential competing events, a Fine-Gray subdistribution hazard model was performed as a sensitivity analysis, considering ACM as the event of interest and kidney transplantation or conversion to peritoneal dialysis as competing events. Missing data rates for HDL-C, LDL-C, and BMI ranged from 5.4 to 11.2%, below the predetermined threshold of 12%; therefore, complete-case analysis was adopted for primary analyses. In addition, multiple imputation was performed for missing variables, and sensitivity analyses were performed to evaluate the impact of missing data on the robustness of the results. All statistical tests were performed using SPSS software (version 26.0) and R software (version 4.2.2). A two-sided *p*-value of less than 0.05 indicated statistical significance (*, *p* < 0.05; **, *p* < 0.01; ***, *p* < 0.001).

## Results

**Figure 1:**
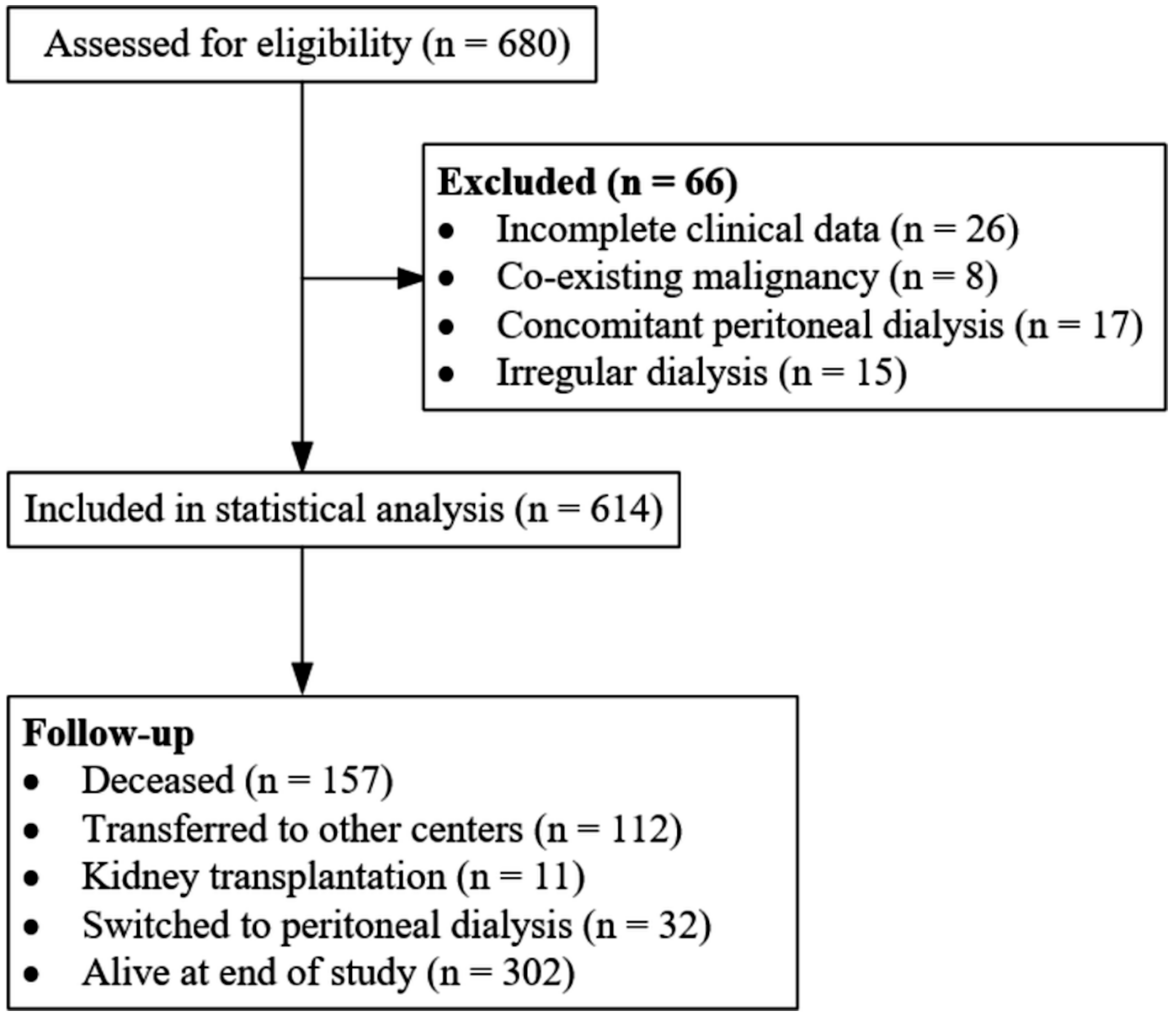
Flowchart of study enrollment.

A total of 680 patients were initially considered for inclusion. However, 66 patients were subsequently excluded for various reasons: incomplete clinical records (*n* = 26), presence of malignancy (*n* = 8), concurrent peritoneal dialysis (*n* = 17), and irregular hemodialysis schedules (*n* = 15). Therefore, 614 patients remained in the final cohort for analysis (Figure 1). As summarized in Table 1, males constituted the majority (65.8%) of the patient group. Hypertensive nephropathy constituted the predominant cause (56.2%) among ESRD etiologies, with diabetic nephropathy (23.3%), chronic glomerulonephritis (17.3%), and polycystic kidney disease (3.3%) subsequently ranked. In terms of vascular access, arteriovenous fistulas (AVFs) were utilized by 80.8% of patients, while CVCs served as the access method in 19.1% of the cohort.

**Table 1:** Demographic characteristics and laboratory parameters of MHD patients according to survival status.

Variables	Total (*n* = 614)	Survivors (*n* = 457)	Non-survivors (*n* = 157)	*p*-value
Age	68.34 ± 10.69	68.25 ± 10.29	68.60 ± 11.81	0.728
Gender				
Male	404 (65.8)	295 (64.6)	109 (69.4)	0.267
Female	210 (34.2)	162(35.4)	48 (30.6)	
BMI	22.56 ± 3.78	22.59 ± 3.84	22.48 ± 3.57	0.786
Dialysis vintage	79.65 ± 43.81	87.64 ± 41.54	56.41 ± 42.05	<0.001^***^
Vascular access				
AVF	497 (80.9)	394 (86.2)	103 (65.6)	<0.001^***^
CVC	117 (19.1)	63 (13.9)	54 (34.4)	
Primary disease				
Chronic glomerulonephritis	106 (17.3)	75 (16.4)	31 (19.7)	
Hypertensive nephropathy	345 (56.2)	277 (60.6)	68 (43.3)	0.015^*^
Diabetic nephropathy	143 (23.3)	93 (20.4)	50 (31.8)	
Polycystic kidney disease	20 (3.3)	12 (2.26)	8 (5.1)	
Laboratory indicators				
AISI	312.86 ± 238.52	364.60 ± 178.78	453.35 ± 321.62	<0.001^***^
CONUT	2.92 ± 1.52	2.64 ± 1.41	3.76 ± 1.52	<0.001^***^
MONO	0.46 ± 0.19	0.44 ± 0.17	0.52 ± 0.21	<0.001^***^
LYM	1.41 ± 0.93	1.51 ± 1.03	1.14 ± 0.50	<0.001^***^
NEU	4.38 ± 1.45	4.22 ± 1.39	4.83 ± 1.52	<0.001^***^
WBC	6.29 ± 1.78	6.16 ± 1.72	6.67 ± 1.92	0.002^**^
PLT	174.05 ± 53.96	174.89 ± 55.10	171.59 ± 50.58	0.508
HGB	105.52 ± 15.19	107.66 ± 14.42	99.28 ± 15.69	<0.001^***^
K^+^	4.87 ± 0.53	4.90 ± 0.51	4.80 ± 0.59	0.049^*^
Ca^2+^	2.27 ± 0.14	2.77 ± 0.14	2.26 ± 0.15	0.137
P	1.74 ± 0.32	1.76 ± 0.29	1.65 ± 0.39	<0.001^***^
TC	3.67 ± 0.96	3.76 ± 0.95	3.41 ± 0.96	<0.001^***^
HDL-C	1.04 ± 0.38	1.06 ± 0.31	0.96 ± 0.53	0.003^**^
LDL-C	1.93 ± 0.72	1.95 ± 0.71	1.88 ± 0.75	0.348
Fe	12.27 ± 3.64	12.61 ± 3.27	11.24 ± 4.43	<0.001^***^
TBIL	7.67 ± 4.50	7.64 ± 4.35	7.75 ± 4.93	0.802
DBIL	2.86 ± 2.19	2.76 ± 1.87	3.16 ± 2.93	0.047^*^
IBIL	4.59 ± 1.76	4.68 ± 1.62	4.34 ± 2.10	0.037^*^
ALT	12.83 ± 11.80	12.13 ± 4.80	14.85 ± 21.77	0.013^*^
AST	18.01 ± 8.42	17.22 ± 5.88	20.31 ± 13.06	<0.001^***^
Urea	20.15 ± 5.12	20.55 ± 4.91	18.99 ± 5.56	0.001^**^
Cr	900.40 ± 539.18	945.15 ± 603.08	770.14 ± 237.23	<0.001^***^
UA	368.51 ± 77.26	373.56 ± 77.59	353.79 ± 74.58	0.005^**^
Alb	39.36 ± 3.50	40.20 ± 3.07	36.92 ± 3.53	<0.001^***^

*p < 0.05; **p < 0.01; ***p < 0.001.

Throughout the observation period, mortality occurred in 157 participants. Leading causes included cardiovascular-related incidents (*n* = 68), cerebrovascular accidents (*n* = 31), infectious diseases (*n* = 19), accidental trauma (*n* = 7), multi-organ dysfunction (*n* = 5), and gastrointestinal bleeding (*n* = 4). Additionally, 23 individuals died outside medical facilities, with unknown exact causes. Compared to survivors, those who died displayed a notably higher rate of CVC usage (*p* < 0.001). Furthermore, deceased participants exhibited significantly decreased concentrations of serum potassium (K^+^), serum calcium (Ca^2+^), TC, high-density lipoprotein cholesterol (HDL-C), serum phosphorus (P), lymphocyte counts (LYM), hemoglobin (HGB), serum iron (Fe), indirect bilirubin (IBIL), serum creatinine (Cr), serum urea, uric acid (UA), and serum Alb (all *p* < 0.05). Conversely, deceased patients showed markedly elevated absolute neutrophil counts (NEU), white blood cell counts (WBC), absolute monocyte counts (MONO), direct bilirubin (DBIL), alanine aminotransferase (ALT), AST, as well as higher CONUT and AISI scores (all *p* < 0.05). Other biochemical markers did not demonstrate statistically significant differences between deceased and surviving patients.

### Predictive performance of AISI and CONUT score for outcomes in MHD patients

**Figure 2:**
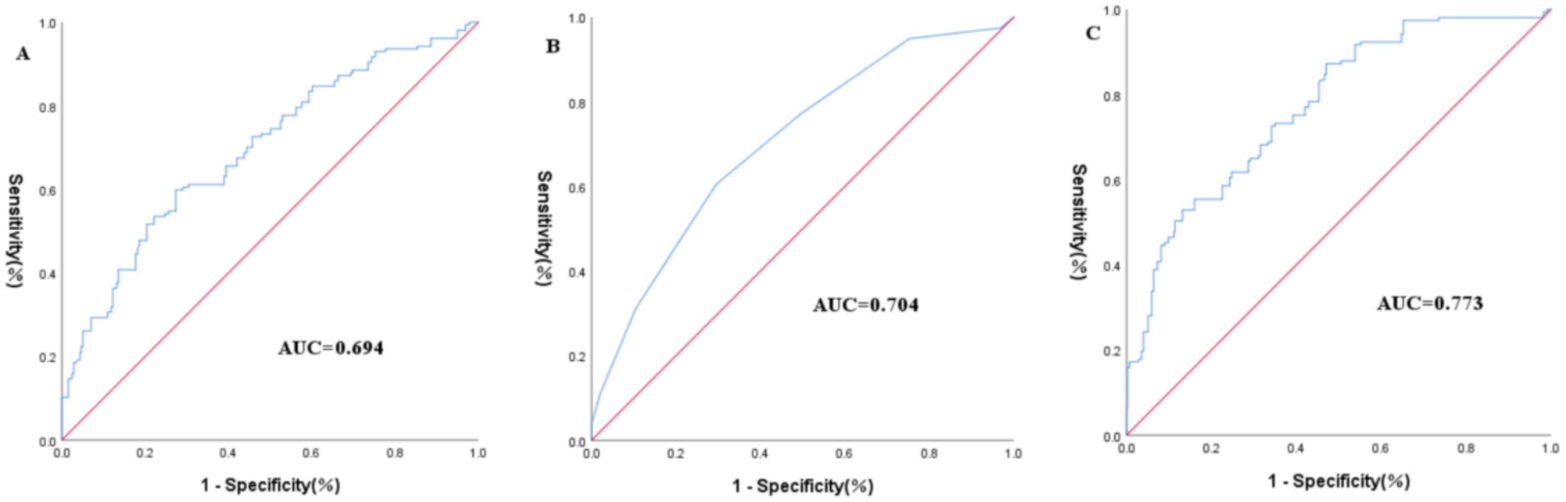
ROC curve analysis of **(A)** AISI, **(B)** CONUT score, and **(C)** AISI+CONUT score in MHD patients.

ROC curves served as a tool to assess the predictive capacity of AISI and CONUT scores concerning outcomes in patients receiving MHD (Figure 2 and Table 2). Figure 2A illustrates that the AUC for AISI was 0.694 (95%CI: 0.645–0.743; *p* < 0.001). In contrast, Figure 2B shows that the AUC for the CONUT score was 0.704 (95%CI: 0.658–0.751; *p* < 0.001), with optimal cutoff values determined to be 306.100 and 3.5, respectively. The integrated model that incorporates AISI and CONUT (Figure 2C) demonstrated an AUC of 0.773 (95% CI, 0.732–0.815; *p* < 0.001). Bootstrap internal validation was performed using 1,000 resamples. The bootstrap-validated AUCs for the AISI, CONUT score, and the combined model were 0.694 (0.632–0.755), 0.704 (0.642–0.762), and 0.772 (0.768–0.774), respectively. These results were consistent with the original AUC estimates, confirming the stability of their discriminatory performance (Table 2).

**Table 2:** ROC analysis and bootstrap internal validation of AISI, CONUT score, and their combination for predicting ACM in MHD patients.

Variables	Original AUC (95%CI)	Bootstrap-validated AUC (95%CI)	Sensitivity (%)	Specificity (%)	Youden index	Cutoff value	p value
AISI	0.694 (0.645–0.743)	0.694 (0.632–0.755)	59.9	72.6	0.325	306.10000	<0.001
CONUT	0.704 (0.658–0.751)	0.704 (0.642–0.762)	60.5	70.5	0.310	3.5	<0.001
AISI+CONUT	0.773 (0.732–0.815)	0.772 (0.768–0.774)	87.3	53.0	0.402	0.162	<0.001

### Kaplan–Meier curves for ACM in MHD patients

**Figure 3:**
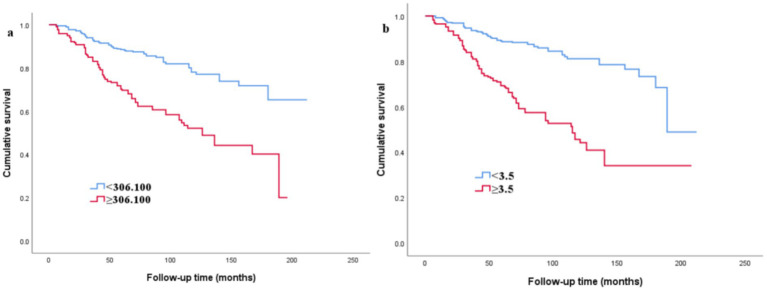
Kaplan–Meier curves for ACM among MHD patients grouped by: **(a)** AISI and **(b)** CONUT.

Based on an optimal cutoff value of 306.100, patients were categorized into high-AISI (≥306.100, *n* = 219) and low-AISI (<306.100, *n* = 395) groups. Patients in the high-AISI group experienced higher rates of ACM (*p* < 0.05). Kaplan–Meier analysis indicated significantly lower cumulative survival in patients with elevated AISI (log-rank *χ*^2^ = 48.085, *p* < 0.001; Figure 3A).

Similarly, patients were categorized into two groups: 230 patients scoring 3.5 or above were classified as the elevated-CONUT group, while 384 patients with scores below 3.5 formed the reduced-CONUT group. The elevated-CONUT group demonstrated significantly increased ACM rates (*p* < 0.05). Lower survival probabilities corresponded to increased CONUT scores, as illustrated in Figure 3B (log-rank *χ*^2^ = 65.147, *p* < 0.001).

### Multivariable-adjusted RCSs

**Figure 4:**
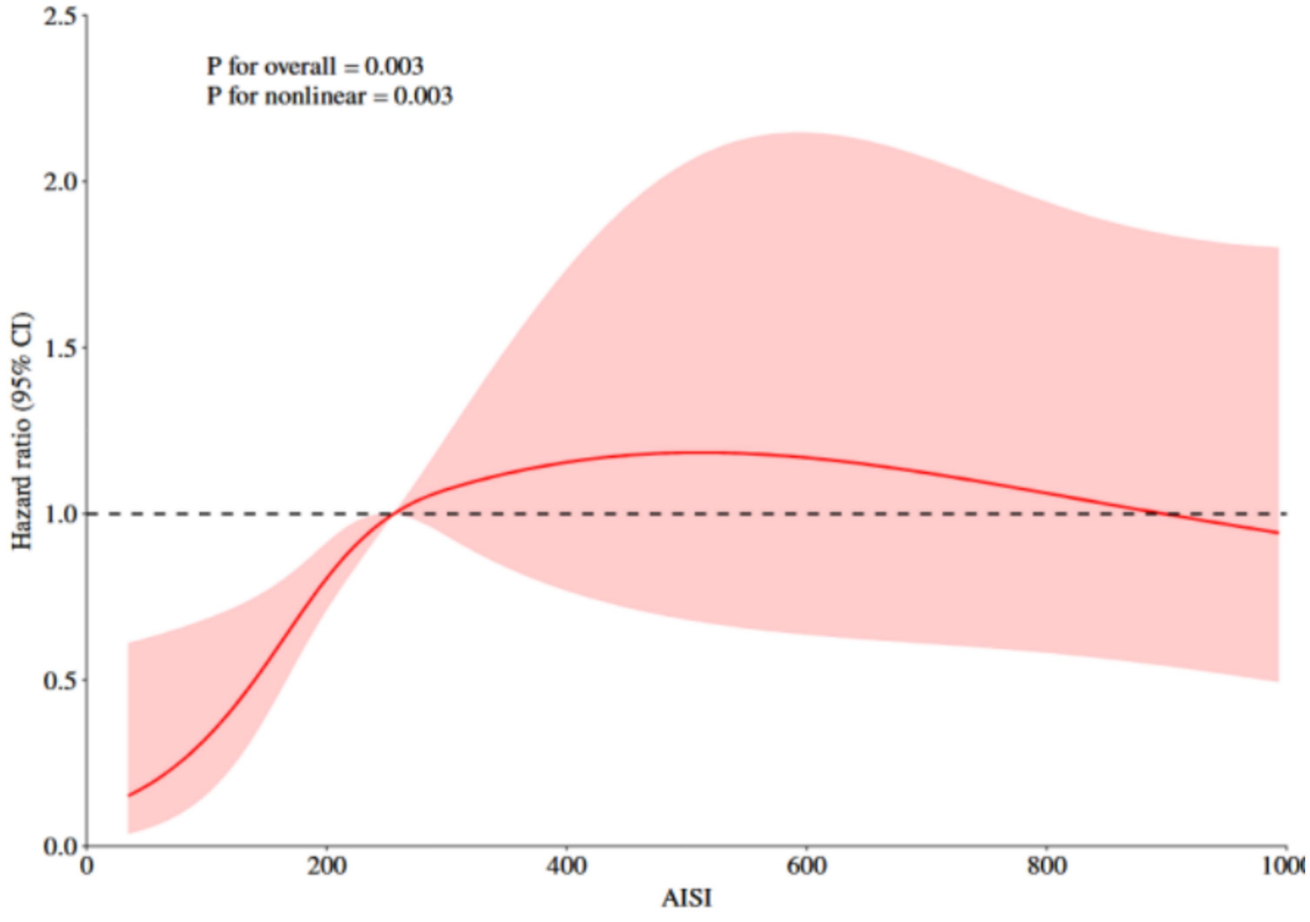
Restricted cubic spline analysis of AISI and risk of ACM.

After adjustment for possible confounders, a nonlinear dose–response relationship between AISI and ACM emerged (p for non-linearity = 0.003). Figure 4 depicts a sharp elevation in mortality risk at lower AISI levels, which plateaued at higher values.

### Identification of independent risk factors

Several factors were significantly linked to ACM in univariate analysis (Table 3, all *p* < 0.05). Prior to constructing the multivariable Cox regression model, multicollinearity diagnostics were conducted. VIF ranged from 1.031 to 1.740, and tolerance values ranged from 0.575 to 0.970, indicating no substantial multicollinearity among the variables included in the final model.

**Table 3:** Univariate and multivariable Cox regression analyses of prognostic factors for ACM.

Variables	Univariate		Multivariate	
	HR (95%CI)	*p*-value	HR (95%CI)	*p*-value
Gender (female vs. male)	0.674 (0.479 ~ 0.948)	0.024	0.459(0.285 ~ 0.739)	0.001^**^
Primary disease (hypertensive nephropathy vs. chronic glomerulonephritis)	0.518 (0.337 ~ 0.795)	0.003		
Primary disease (diabetic nephropathy vs. chronic glomerulonephritis)	1.160 (0.738 ~ 1.823)	0.520		
Primary disease (polycystic kidney disease vs. chronic glomerulonephritis)	0.979 (0.449 ~ 2.135)	0.956		
Vascular access (CVC VS. AVF)	2.708 (1.947 ~ 3.766)	<0.001^***^	1.991(1.339 ~ 2.961)	0.001^**^
Laboratory indicators				
CONUT(≥3.5 vs. <3.5)	3.463 (2.509 ~ 4.780)	<0.001^***^	3.574(2.479 ~ 5.153)	<0.001^***^
AISI(≥306.100 vs. <306.100)	2.923 (2.124 ~ 4.024)	<0.001^***^	2.622(1.708 ~ 4.026)	<0.001^***^
MONO	3.318 (2.181 ~ 5.049)	<0.001^***^		
LYM	0.477 (0.339 ~ 0.671)	<0.001^***^		
NEU	1.230(1.119 ~ 1.352)	0.002^**^		
HGB	0.951 (0.940 ~ 0.963)	<0.001^***^	0.971(0.957 ~ 0.986)	<0.001^***^
K^+^	0.649 (0.472 ~ 0.891)	0.008^**^		
Ca^2+^	0.201 (0.069 ~ 0.590)	0.003^**^		
P	0.285 (0.164 ~ 0.495)	<0.001^***^		
TC	0.641 (0.529 ~ 0.776)	<0.001^***^		
HDL-C	0.462 (0.267 ~ 0.799)	0.006		
IBIL	0.908 (0.854 ~ 0.965)	0.006^**^		
AST	1.022 (1.013 ~ 1.031)	<0.001^***^	1.032(1.015 ~ 1.049)	<0.001^***^
Urea	0.938 (0.904 ~ 0.973)	0.001^**^	0.955(0.914 ~ 0.998)	0.040^*^
Cr	0.997 (0.996 ~ 0.998)	<0.001^***^	0.998(0.997 ~ 1.000)	0.018^*^
UA	0.996 (0.994 ~ 0.998)	<0.001^***^		
Alb	0.729 (0.691 ~ 0.769)	<0.001^***^		

*p < 0.05; **p < 0.01; ***p < 0.001.

Multivariable Cox regression analysis revealed that CVC use, CONUT score ≥3.5, AISI ≥306.100, and elevated AST levels were independently associated with increased five-year ACM in patients undergoing MHD (all *p* < 0.05). Conversely, female sex and higher levels of hemoglobin, creatinine, and urea were associated with reduced mortality risk (all *p* < 0.05).

### Sensitivity analyses

Multiple-imputation sensitivity analysis. Missing values were limited to BMI (11.2%), HDL-C (5.4%), and LDL-C (5.4%). There were no missing data for survival time, mortality status, AISI, CONUT score, or the main covariates retained in the multivariable Cox regression model. Multiple imputation was performed for variables with missing data, and the multivariable Cox regression analysis was repeated using imputed datasets. Results from the imputed data analysis were consistent with the complete-case analysis: AISI ≥306.100 (HR = 3.55, 95% CI: 2.50–5.05, *p* < 0.001) and CONUT score ≥3.5 (HR = 2.53, 95% CI: 1.66–3.84, *p* < 0.001) remained independently associated with increased five-year ACM. These findings confirm the robustness of the primary results.

Competing-risk sensitivity analysis. Kidney transplantation and conversion to peritoneal dialysis were considered competing events, while ACM was the primary event of interest. Transfer to other facilities and survival until the end of follow-up was treated as censored observations. Cumulative incidence curves showed that patients with AISI ≥306.100 had a higher incidence of ACM than those with AISI <306.100 after accounting for competing risks. Similarly, patients with a CONUT score ≥3.5 exhibited higher cumulative ACM incidence compared with those scoring <3.5. In the Fine-Gray subdistribution hazard model, both AISI ≥306.100 (sHR = 2.530, 95% CI: 1.515–4.227, *p* < 0.001) and CONUT score ≥3.5 (sHR = 2.022, 95% CI: 1.284–3.186, *p* = 0.002) remained significant predictors of ACM. These results were consistent with those obtained from the Cox regression and Kaplan–Meier analyses, further supporting the robustness of the findings.

## Discussion

In this study, CONUT score ≥3.5 and AISI ≥306.100 were independently associated with five-year ACM among patients undergoing MHD, indicating their value as prognostic markers with enhanced predictive accuracy when combined. Bootstrap internal validation confirmed that the validated AUCs closely matched the original ROC results, reinforcing the reliability of these findings. In ESRD patients, inflammation results from a complex interplay among various factors, including exposure to dialysis materials, and uremic toxin accumulation. This chronic inflammatory state significantly worsens patient outcomes, ultimately leading to increased mortality. In clinical practice, dynamic long-term monitoring is frequently required for MHD patients. Although cytokines such as IL-6 are sensitive inflammatory markers, their short half-life, high detection cost, and circadian variability limit their routine applicability in large-scale follow-ups. In contrast, AISI is calculated from inexpensive, routinely available complete blood count data, offering significant economic advantages and enabling clinicians to retrospectively evaluate long-term inflammation. Specifically, increased neutrophil, monocyte, and platelet counts reflect nonspecific inflammatory responses and a prothrombotic state. Conversely, lymphopenia not only indicates impaired immune regulation but also represents malnutrition (12). Therefore, AISI essentially reflects the imbalance between proinflammatory/prothrombotic mechanisms and the immune-nutritional defense in ESRD patients. This cumulative effect enhances its sensitivity to detect micro-inflammation. A prior large-scale cross-sectional analysis leveraging National Health and Nutrition Examination Survey (NHANES) data revealed a robust association between elevated AISI and CKD, signifying greater CKD prevalence with increasing AISI levels (13). Additionally, research examining patients with coexisting CKD and coronary artery disease found that elevated AISI values independently forecasted increased risks of cardiovascular and ACM, underscoring its utility in risk evaluation among CKD patients (14). The present analysis identified an optimal threshold for AISI at 306.100, consistent with previous findings by Chen et al. (15). In MHD patients, oxidative stress and uremic toxins not only trigger systemic inflammation but also cause vascular endothelial injury, accelerating atherosclerosis and increasing cardiovascular risk. Thus, AISI is a sensitive indicator of micro-inflammation and an essential link connecting malnutrition, inflammation, and cardiovascular outcomes.

Protein-energy wasting (PEW) in ESRD patients increases morbidity and mortality. Additionally, malnutrition, inflammation, and other contributing factors further accelerate disease progression, increasing hospitalization rates and impairing quality of life. Previous meta-analytic evidence has demonstrated that malnutrition is a strong determinant of mortality in patients receiving dialysis, with the effect being particularly pronounced among those undergoing MHD (2). Consequently, timely and accurate nutritional evaluation is of critical importance in this population. Kamath et al. first introduced a nutritional screening approach based on serum Alb, LYMs, and TC (16). Expanding upon this idea, the CONUT scoring system was introduced by Ulíbarri et al. in 2005 as a method to screen for malnutrition risk in outpatient populations (17). By integrating laboratory indicators associated with nutrition, immune status, and systemic inflammation, including Alb and LYM, the CONUT score effectively captures nutritional status, with elevated scores indicating diminished nutritional reserves and increased vulnerability. Higher CONUT scores have repeatedly demonstrated strong associations with heightened risks of cardiovascular and ACM. Specifically, severe malnutrition, defined by a CONUT score ≥ 3, was linked to a 2.71-fold increased risk of ACM relative to individuals exhibiting adequate nutrition (18). Moreover, among diabetic nephropathy patients, the CONUT score independently predicted advancement to ESRD, ACM, and cardiovascular incidents (19). Each additional point increment in the CONUT score corresponded to approximately 13, 16, and 30% higher risks for ESRD, cardiovascular incidents, and ACM, respectively (19). Notably, even following multivariate adjustment for known confounders such as age, BMI and diabetes diagnosis, the CONUT score persisted as a strong, independent predictor of mortality risk within dialysis cohorts (20). Serum albumin, a primary component of the CONUT index, is frequently employed as a marker of nutritional adequacy and clinical outcomes in MHD patients (21); however, its levels may be negatively impacted by chronic systemic inflammation (22). Additionally, malnutrition impairs immune function, disrupts lymphocyte maturation, and reduces LYMs, weakening host resistance to infection (7). In this study, the mean CONUT score was 2.92, and the optimal cutoff value was 3.5, categorizing patients into low (≤3) and high (≥4) groups. This finding indicates that when the CONUT score reaches or exceeds 4, patients are already experiencing significant malnutrition and immune dysfunction, substantially increasing the risk of adverse outcomes. Consequently, patients with elevated CONUT scores should undergo early comprehensive nutritional assessments and receive individualized interventions. Such interventions include optimizing protein and energy intake, reducing inflammation, and enhancing infection prevention strategies to lower adverse outcomes and improve long-term prognosis.

Compared with other nutrition- or inflammation-related assessment tools, such as the malnutrition-inflammation score (MIS) and geriatric nutritional risk index (GNRI), the AISI and CONUT score have practical advantages for routine MHD management. MIS comprehensively evaluates malnutrition-inflammation status specifically in dialysis patients, but includes clinical and subjective components, limiting feasibility in retrospective analyses or large-scale screenings. The GNRI, while simple and widely utilized, primarily reflects nutritional status through serum albumin and body weight and does not directly assess systemic inflammation or hematologic immune responses. In contrast, the AISI and CONUT score are based entirely on routinely available laboratory parameters. The AISI assesses systemic inflammation and prothrombotic burden via peripheral blood cell counts, whereas the CONUT score reflects nutritional reserves and immune status through albumin, cholesterol, and lymphocyte counts. Therefore, the combined use of AISI and CONUT provides a simple, objective, and cost-effective approach for identifying MHD patients at risk due to inflammatory and nutritional vulnerabilities.

Clinically, integrating AISI and CONUT may offer complementary risk-stratification information for MHD patients. Elevated AISI predominantly indicates systemic inflammation and prothrombotic status, whereas a high CONUT score identifies nutritional impairment and compromised immune function. Patients exhibiting simultaneous elevations in both indices represent a particularly vulnerable subgroup at heightened risk. In routine practice, a stepwise screening strategy using these indices could efficiently identify high-risk patients: the AISI identifies increased inflammatory burden, and the CONUT score further clarifies nutritional and immune impairment. Although the present study did not establish a formal risk score or nomogram, the combined assessment of AISI and CONUT may help clinicians recognize patients requiring closer monitoring, nutritional assessment, inflammation control, infection prevention, and individualized interventions.

Cox proportional hazards regression analysis identified CVC use, CONUT score ≥3.5, AISI ≥306.100, and elevated AST levels as independent predictors of increased five-year ACM among MHD patients. In contrast, female sex and higher hemoglobin, creatinine, and urea levels were associated with lower mortality risk. Vascular access type is a crucial clinical factor in hemodialysis, significantly influencing patient outcomes. Compared with AVF, CVCs are more frequently utilized in patients with limited vascular access options, delayed planning, or clinically unstable conditions. CVC-based dialysis may elevate infection risks and inflammatory exposure, contributing to poorer prognoses. In this study, the association between CVC use and ACM persisted after adjusting for multiple covariates, suggesting that vascular access type can effectively identify patients with higher overall risk profiles. However, given the observational study design, these associations should be interpreted cautiously and primarily regarded as prognostic within the context of routine MHD clinical management.

The study findings further emphasize that female sex functions as an independent protective factor against five-year ACM, corroborating prior research outcomes (23, 24). Female dialysis patients, compared to their male counterparts, tend to demonstrate lower systemic inflammatory responses, more stable and balanced immune functions, and reduced susceptibility to cardiovascular events. These gender-based physiological and immunological distinctions contribute substantially to the observed survival advantage in female patients undergoing MHD. Moreover, the current study identifies an inverse relationship between lower serum creatinine and urea levels and increased mortality risk, aligning with previous observations (25) and exemplifying the well-documented phenomenon known as “reverse epidemiology” within the dialysis population. Unlike in individuals with normal renal function, serum creatinine concentrations in MHD patients primarily represent skeletal muscle mass rather than renal filtration efficiency. Consequently, creatinine levels act as an indirect indicator of somatic protein reserves and muscle health (26). Higher serum creatinine typically denotes superior muscle mass and enhanced nutritional status, enabling improved patient resilience to infections, inflammatory insults, and catabolic stress conditions. Analogously, elevated serum urea levels frequently indicate sufficient dietary protein intake, maintaining positive nitrogen balance, and reflecting the absence of significant protein-energy wasting (27). Thus, moderate elevations in serum creatinine and urea concentrations collectively indicate more favorable nutritional, metabolic, and immunological conditions, contributing to improved survival outcomes in MHD patients.

Under normal physiological circumstances, serum AST activity typically remains low among patients undergoing MHD. Elevated AST levels, however, often signify hepatic impairment, tissue ischemia, or an elevated inflammatory state, indicating potential underlying pathology. This notion aligns with findings reported by Xue et al. (25) who demonstrated that increased serum AST levels independently forecast higher mortality in MHD patients. This prognostic relationship was further reinforced by the current study’s findings, emphasizing AST’s clinical relevance as a significant biomarker in predicting patient outcomes. Conversely, ALT did not exhibit a statistically significant association with mortality risk in this patient population. This discrepancy can likely be attributed to the distinct tissue distributions of these enzymes. AST is widely distributed across various tissues, including liver, skeletal muscle, cardiac muscle, kidneys, and brain, whereas ALT is predominantly localized within hepatocytes. Consequently, elevated AST levels reflect broader tissue injury or systemic inflammatory responses, thus better capturing the overall health status and mortality risk among MHD patients compared to ALT.

## Limitations

This study has several limitations. First, it was a retrospective, single-center study with a relatively modest sample size, potentially limiting the generalizability of the findings. Although patients were recruited from three hemodialysis units within the same institution, variations in patient characteristics, dialysis practices, and clinical management across centers might restrict the applicability of our results to broader MHD populations. Therefore, prospective, multicenter studies with larger sample sizes are required to validate the prognostic utility of the AISI and CONUT score further.

Second, residual confounding cannot be completely excluded. While multivariable Cox regression adjusted for available clinical and laboratory covariates, several potentially relevant factors, such as dialysis adequacy parameters, detailed comorbidity burden, medication history, frailty status, baseline cardiac function, and socioeconomic factors, were not comprehensively captured in this retrospective dataset. Although CVC use was independently associated with ACM in the multivariable model, residual confounding related to vascular access selection factors such as vascular condition, urgency of dialysis initiation, suitability for AVF creation, and comorbidity burden might remain. Therefore, the association between CVC use and mortality should be interpreted within the broader context of clinical risk heterogeneity.

Third, although time-averaged laboratory values were used to better reflect long-term inflammatory and nutritional status, this method cannot fully capture dynamic longitudinal changes in AISI and CONUT scores. Adjustments in nutritional supplementation, dialysis prescriptions, medications, infection prevention strategies, and individualized clinical management might affect these indicators over time. Future studies using time-dependent Cox models or trajectory-based analytical approaches is necessary to clarify the prognostic implications of longitudinal variations in AISI and CONUT scores.

Fourth, potential selection bias arising from data handling should be acknowledged. Laboratory results obtained during hospitalization or active infection were excluded to reduce the influence of acute-phase inflammatory responses; however, this exclusion criterion could omit data from patients with more severe clinical conditions or recurrent infections. In addition, missing data were limited to BMI, HDL-C, and LDL-C, and multiple-imputation sensitivity analyses yielded consistent results with the complete-case analyses, biases associated with incomplete clinical records remain a concern due to.

Finally, although bootstrap internal validation was performed to confirm the stability of the AUC estimates, external validation using independent cohorts was unavailable. Optimal cutoff values for the AISI and CONUT score identified in this study are dataset-specific and must be cautiously interpreted. Further validation in independent, multicenter cohorts is required before these thresholds can be broadly applied clinically.

## Conclusion

In conclusion, this study demonstrates that elevated AISI and CONUT scores are significantly associated with increased mortality risk among patients undergoing MHD. Both indices utilize routinely available laboratory parameters, making them practical and accessible tools for patient monitoring and risk stratification in clinical practice. Multivariable analysis identified CVC use, AISI ≥306.100, CONUT score ≥3.5, and elevated AST levels as independent predictors of increased five-year ACM, whereas female sex and higher hemoglobin, urea, and creatinine levels were associated with reduced mortality risk. Collectively, these findings support the clinical value of the AISI and CONUT score as simple, low-cost, and objective prognostic indicators to identify high-risk MHD patients who may benefit from enhanced monitoring and individualized management.

## Data Availability

The raw data supporting the conclusions of this article will be made available by the authors, without undue reservation.
